# The Behavior of Amphibians Shapes Their Symbiotic Microbiomes

**DOI:** 10.1128/mSystems.00626-20

**Published:** 2020-07-28

**Authors:** Liangliang Xu, Mengmeng Xiang, Wei Zhu, Mengjie Zhang, Hua Chen, Jin Huang, Youhua Chen, Qing Chang, Jianping Jiang, Lifeng Zhu

**Affiliations:** a Chengdu Institute of Biology, Chinese Academy of Sciences, Chengdu, China; b College of Life Sciences, Nanjing Normal University, Nanjing, China; c Miao Tong (Shang Hai) Biological & Technology Co. Ltd., Shanghai, China; d Mingke Biotechnology Co., Ltd., Hangzhou, China; e Shengda Hydropower Co., Ltd., Sinohydro Group Ltd., Leshan, China; University of California San Diego

**Keywords:** seasonal dynamics, symbiotic microbiome, poikilothermic animals, behavior, living environment

## Abstract

Seasonal dynamics in symbiotic microbiomes have been investigated in a number of vertebrates and are mainly caused by changes in the diet (in the gut microbiome) or the living environment (in the skin microbiome). Most amphibian microbiome studies focus on the skin, whereas internal microbiome structure and dynamics are often overlooked. The present study investigated the seasonal dynamics in three types of symbiotic microbiomes (the skin, stomach, and gut) across four wild frog species, belonging to different families, in May and October. The frogs harbored more water source microbes in May than in October. On the contrary, the frogs harbored more soil source microbes in October than in May. The frog species investigated tend to live in a water environment in May to maintain body surface humidity at high environmental temperatures and to breed. In October, these four species prefer to live on the land, as the environmental temperature decreases, to prepare for hibernation in caves or under stones. Thus, seasonal changes in the wild amphibian symbiotic microbiome may be caused by the difference in microbe transmission from their living environment due to specific behaviors. This study demonstrated that the behavior and living environment of wild amphibians shape their symbiotic microbiome externally (on the skin) and internally (in the stomach and gut). We revealed the potential association between specific behaviors in poikilothermic animals and host symbiotic microbiomes.

**IMPORTANCE** Understanding the interactions between host behavior and microbiome dynamics remains an outstanding priority in the field of microbial ecology. Here, we provide the reader with a simple example of how the behavior and living environment of wild amphibians shape their symbiotic microbiome externally (on the skin) and internally (in the stomach and gut).

## Abstract (precis)

Understanding the interactions between host behavior and microbiome dynamics remains an outstanding priority in the field of microbial ecology. Here, we provide the reader with a simple example of how the behavior and living environment of wild amphibians shape their symbiotic microbiome externally (on the skin) and internally (in the stomach and gut).

## INTRODUCTION

Amphibians, as poikilothermic animals, are very sensitive to changes in the natural environment and can regulate their behavior, for example, through hibernation, to maintain the optimum temperature for growth and development (1, 2). When the temperature rises, amphibians such as Ambystoma opacum migrate to ponds or wetlands to breed, to ensure the survival of their offspring (3–5). When the temperature decreases, amphibians migrate to hibernation sites to maintain their body temperature during the cold winter (6).

In amphibians, skin microbes are sensitive to environmental changes, such as temperature and moisture (7, 8). Microbial transmission occurs between the skin microbes of amphibians and the environment (9, 10). For example, the European common frog (Rana temporaria) living in complex habitats with higher environmental bacterial species richness harbored greater mean skin bacterial diversity than that living in the simple habitat (10). Another study reported minimal overlap between amphibian core microbes and the most abundant environmental microbes (9). The skin microbes of juvenile bullfrogs (Rana catesbeiana) and adult red-spotted newts (Notophthalmus viridescens) and environmental microorganisms in substrate and water account for only a small part of the environmental microbes (<66%) (9). Moreover, environmental changes affect the gut microbiome (11, 12). In reptiles such as Liolaemus parvus, Liolaemus ruibali, and Phymaturus williamsi, the relative abundance of fecal microbes of the offspring overlaps with maternal microbes, due to gut microbial transmission during birth (vertical transmission) (13). Horizontal transmission in lizards occurs through close association with conspecifics, dietary sources, living environment, and coprophagy (14). There is a growing interest in the notion that behavioral processes serve as significant predictors of the similarities and differences in the gut microbial organization (15). The transmission of gut microbes has been demonstrated or implicated in several species, including bumblebees (16), zebra finches (17), ponies (18), baboons (19), sifakas (20), humans, and domestic dogs (21). However, in amphibians, the effect of specific behaviors to adapt to environmental changes, such as temperature changes, on the symbiotic microbiomes (for example, on the skin and in the gut) have not been well investigated.

**FIG 1:**
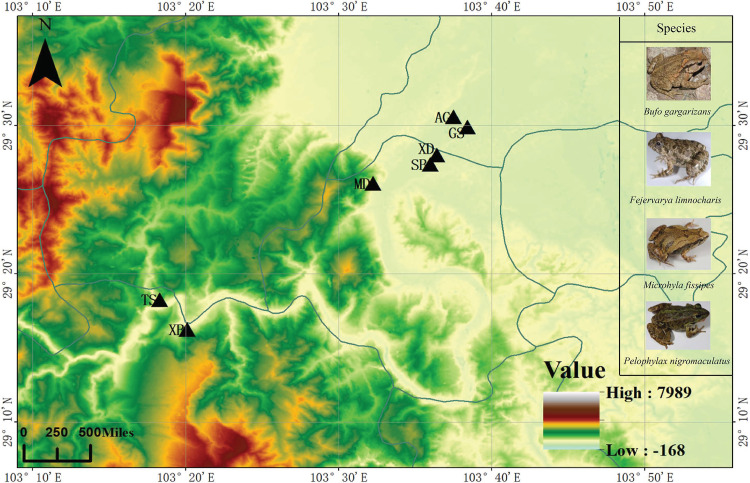
Study area including the sampling sites for the four frog species investigated. The black triangles represent the sampling sites. GS, Gaoshan country; XD, Xiaodian country; SB, Sunba country; MD, Muodong country; TS, Tongshan country; XB, Xuba country; AG, Ange country.

The present study investigated how specific behaviors of four wild frog species Bufo gargarizans (Bufonidae), Fejervarya limnocharis (Dicroglossidae), Pelophylax nigromaculatus (Ranidae), and Microhyla fissipes (Microhylidae) affect their symbiotic microbiome (on the skin and in the stomach and gut) in May and October (Fig. 1; see also Table S1 in the supplemental material). In May, these four frog species prefer to live in a water environment to maintain body surface humidity at high environmental temperatures and to breed. B. gargarizans is one kind of terrestrial amphibian and prefers to select bare surfaces or low vegetable as feeding sites in southwestern China (22). In October, when the environmental temperature decreases, these four frog species prefer to live on land. The hibernation period of these species lasts between late September and late March (23–25); hibernation sites include caves and stones, and P. nigromaculatus also selects the sludge at the bottom (23, 24). Importantly, frogs have a similar diet, consisting of insects such as Hymenoptera and Coleoptera, during different seasons (25–28).

10.1128/mSystems.00626-20.4 TABLE S1 Summary of the characteristics of the samples obtained from the frogs at different sampling times. Species (n1, n2, n3) represented the number of skin, stomach, and gut samples, respectively. GS, Gaoshan; XD, Xiaodian; SB, Sunba; MD, Muodong; TS, Tongshan; XB, Xuba; AG, Ange. Altitude is presented in meters (m). Download Table S1, DOCX file, 0.01 MB. Copyright © 2020 Xu et al. 2020 Xu et al. This content is distributed under the terms of the Creative Commons Attribution 4.0 International license.

Therefore, the present study focused on the potential differences in the symbiotic microbiomes on the skin and in the stomach and gut in the four frog species between May and October due to two types of behaviors: water and land living. We hypothesized that in May, the proportion of the symbiotic microbes from water source microbes would be higher than in October and that in October, the proportion of the symbiotic microbes from soil source microbes would be higher than in May. First, we wanted to verify the common pattern of microbiome transmission among these different species and did not compare the difference in the microbiome compositions across different species or among different types of symbiotic microbiomes. Second, considering the strength of the relationship between environment and microbiome to vary in concert with species due to host behavior, we discuss the host species differences on the microbiome transmission in this study, especially in the skin microbiome (directly contacting the living environment).

## RESULTS

### Changes in the symbiotic microbiome between May and October.

Linear discriminant analysis effect size (LEfSe) revealed a putative consensus pattern in the changes of the gut microbiome across the four frog species (see Table S2 in the supplemental material). For example, the abundance of *Actinobacteria* was significantly higher in skin samples obtained in October than in those obtained in May. The abundance of *Sphingomonadaceae* (*Proteobacteria*) was significantly higher in skin samples obtained in May than in those obtained in October. The abundance of *Clostridiaceae* 1 (*Firmicutes*) was significantly higher in gut samples obtained in May than in those obtained in October. In soil samples, the abundance of *Firmicutes* was significantly higher in May than in October, and the abundance of *Cyanobacteria* and *Bacteroidetes* was significantly higher in October than in May (Fig. S1). In water samples, the abundance of *Proteobacteria* and *Actinobacteria* was significantly higher in May than in October (Fig. S2).

10.1128/mSystems.00626-20.1 FIG S1 Linear discriminant analysis effect size (LEfSe) in the soil microbiome community between May and October samples. Download FIG S1, DOCX file, 0.3 MB. Copyright © 2020 Xu et al. 2020 Xu et al. This content is distributed under the terms of the Creative Commons Attribution 4.0 International license.

10.1128/mSystems.00626-20.2 FIG S2 Linear discriminant analysis effect size (LEfSe) in the water microbiome community between May and October samples. Download FIG S2, DOCX file, 0.3 MB. Copyright © 2020 Xu et al. 2020 Xu et al. This content is distributed under the terms of the Creative Commons Attribution 4.0 International license.

10.1128/mSystems.00626-20.5 TABLE S2 Significant difference in the abundance of symbiotic microbiomes (at the phylum and family levels) in the four frog species between May and October samples using the linear discriminant analysis (LDA) effect size (LEfSe) method. The bold font represents the significance in the abundance of this microbe between the May and October samples. Download Table S2, DOCX file, 0.01 MB. Copyright © 2020 Xu et al. 2020 Xu et al. This content is distributed under the terms of the Creative Commons Attribution 4.0 International license.

**FIG 2:**
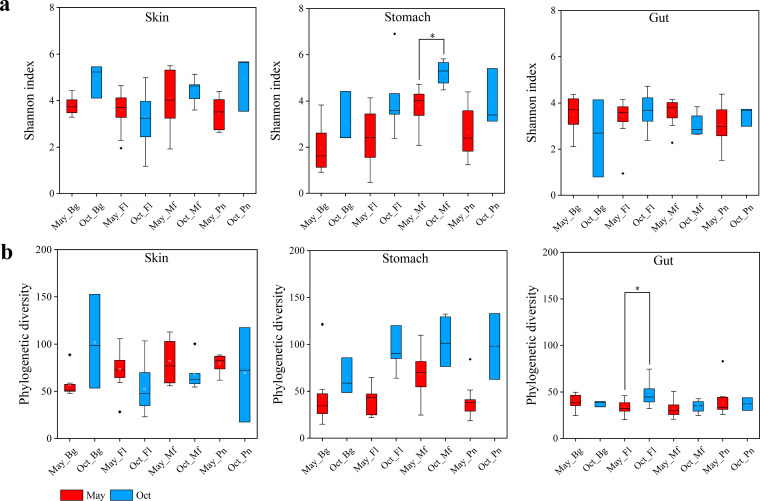
Box plots of alpha diversity of the symbiotic microbiomes in the four frog species. (a) Shannon index comparisons for the skin, stomach, and gut microbes between May and October (Oct) samples in each frog species. (b) Phylogenetic diversity comparisons for the skin, stomach, and gut microbes between May and October samples in each frog species. Bg, *Bufo gargarizans* samples; Fl, *Fejervarya limnocharis* samples; Mf, *Microhyla fissipes* samples; Pn, *Pelophylax nigromaculatus* samples. The Mann-Whitney *U* test was used to analyze the differences between the May and October samples in each type of symbiotic microbiome within the same frog species. *, *P* < 0.002 after the Dunn-Sidàk correction. The upper and lower whiskers represent scores outside the middle 50% (i.e., the lower 25% of scores and the upper 25% of scores). The minimum score is the lowest score, excluding outliers (shown at the end of the down whisker). The maximum score is the highest score, excluding outliers (shown at the end of the top whisker). In the boxes, the upper lines represent the upper quartiles (75th percentiles), the bottom lines represent the lower quartiles (25th percentiles), the lines between the upper and bottom lines represent the median values, and the squares represent mean values. The black diamonds represent the outliers.

There were no consensus changes in the alpha diversity (Shannon index and phylogenetic diversity) in the symbiotic microbiomes between May and October, with the exception of the phylogenetic diversity in stomach samples (Fig. 2a and b). The phylogenetic diversity was higher in the October samples of three of the four frog species than in those in May.

### Dissimilarity in the symbiotic microbiome community between May and October.

**FIG 3:**
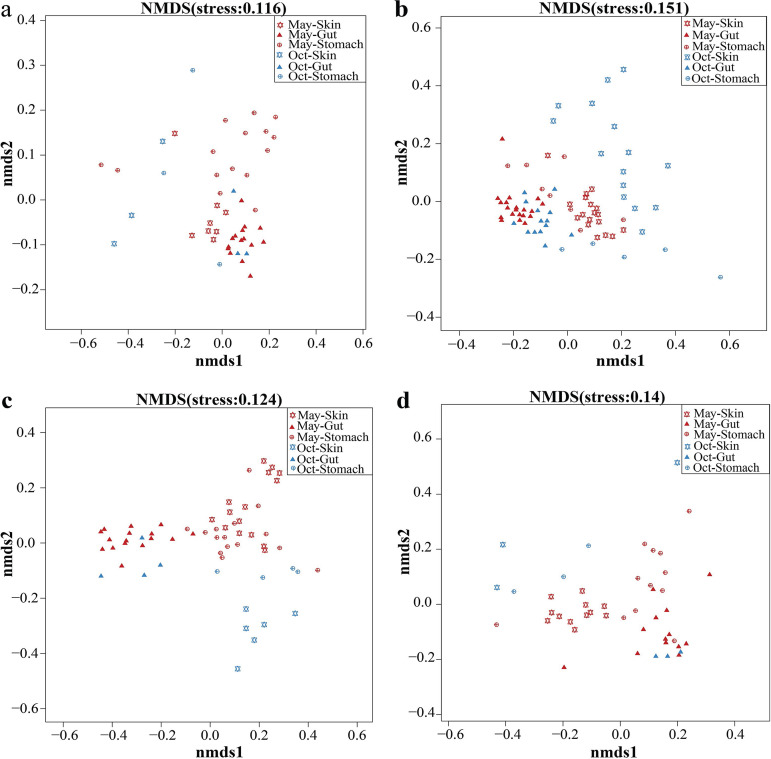
Nonmetric multidimensional scaling analysis using unweighted UniFrac distance revealed the dissimilarity in the symbiotic microbiome communities between May and October (Oct) for each frog species. (a) *Bufo gargarizans* samples. (b) *Fejervarya limnocharis* samples. (c) *Microhyla fissipes* samples. (d) *Pelophylax nigromaculatus* samples.

Adonis results (see Table S3) from a permutational multivariate analysis of variance (PERMANOVA) revealed that there was a significant dissimilarity in each type of symbiotic microbiome community in the frog, soil, and water samples between May and October. Adonis was used to compute an *R*^2^ value, which showed the percentage of seasonal variation (May and October). In most of the frog species, this dissimilarity was larger in the skin microbiome than in the stomach and gut microbiomes. For example, the *R*^2^ values were 0.299, 0.083, and 0.114 in B. gargarizans skin, stomach, and gut samples, respectively (Table S3). The gut microbiome showed the lowest distance (unweighted UniFrac) between May and October in three of four frog species compared with those in the skin and stomach microbiomes (Fig. S3). Nonmetric multidimensional scaling (NMDS) analysis further revealed this dissimilarity (Fig. 3).

10.1128/mSystems.00626-20.3 FIG S3 Unweighted UniFrac distance of skin, stomach, and gut microbes of amphibians between May and October. Black dots represent outliers. The Mann-Whitney *U* test was used to test the differences between skin, stomach, and gut within one frog species, NS, *P > *0.05; *, *P* < 0.05; ***, *P* < 0.001; ****, *P* < 0.0001. For example, skin represented the pairwise comparison between May skin samples from one frog species with October skin samples from this one frog species. Bg, the samples from *Bufo gargarizans*; Fl, the samples from *Fejervarya limnocharis*; Mf, the samples from *Microhyla fissipes*; Pn, the samples from *Pelophylax nigromaculatus*. Download FIG S3, DOCX file, 0.09 MB. Copyright © 2020 Xu et al. 2020 Xu et al. This content is distributed under the terms of the Creative Commons Attribution 4.0 International license.

10.1128/mSystems.00626-20.6 TABLE S3 Adonis results (PERMANOVA) of each type of the symbiotic microbiomes within each species in May and October samples. There were two groups (the samples in May and the samples in October) for the comparison in each type of the symbiotic microbiome within each species. Thus, the *Df* value was one. Bg, *Bufo gargarizans*; Fl, *Fejervarya limnocharis*; Mf, *Microhyla fissipes*; Pn, *Pelophylax nigromaculatus*. Download Table S3, DOCX file, 0.01 MB. Copyright © 2020 Xu et al. 2020 Xu et al. This content is distributed under the terms of the Creative Commons Attribution 4.0 International license.

In addition, we found dissimilarity in the microbiome communities on the skin and in the stomach, gut, water, and soil samples (Fig. 3) between May and October. There was significant dissimilarity in each type of symbiotic microbiome among the different frog species (Table S4).

10.1128/mSystems.00626-20.7 TABLE S4 Adonis results (PERMANOVA) of the symbiotic microbiomes among four frog species. Download Table S4, DOCX file, 0.01 MB. Copyright © 2020 Xu et al. 2020 Xu et al. This content is distributed under the terms of the Creative Commons Attribution 4.0 International license.

### Putative contributions of the living environmental microbiome to the amphibian symbiotic microbiomes in May and October.

**FIG 4:**
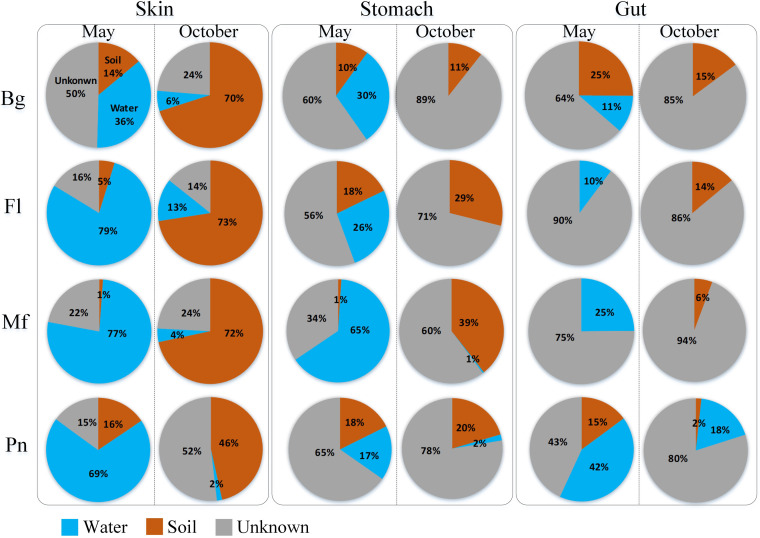
Source-tracking analysis showing the mean putative contributions of the environmental microbiomes to the symbiotic microbiomes of each frog species in May and October. The blue, brown, and gray colors indicated water source, soil source, and unknown source microbiomes, respectively. Water source microbiome in the host symbiotic microbiome meant that the host likely acquired the microbiome from the aquatic environment. Soil source microbiome in the host symbiotic microbiome meant that the host likely acquired the microbiome from the land environment. Each row shows the analyses based on the samples from one kind of frog species. Bg, *Bufo gargarizans* samples; Fl, *Fejervarya limnocharis* samples; Mf, *Microhyla fissipes* samples; Pn, *Pelophylax nigromaculatus* samples.

**FIG 5:**
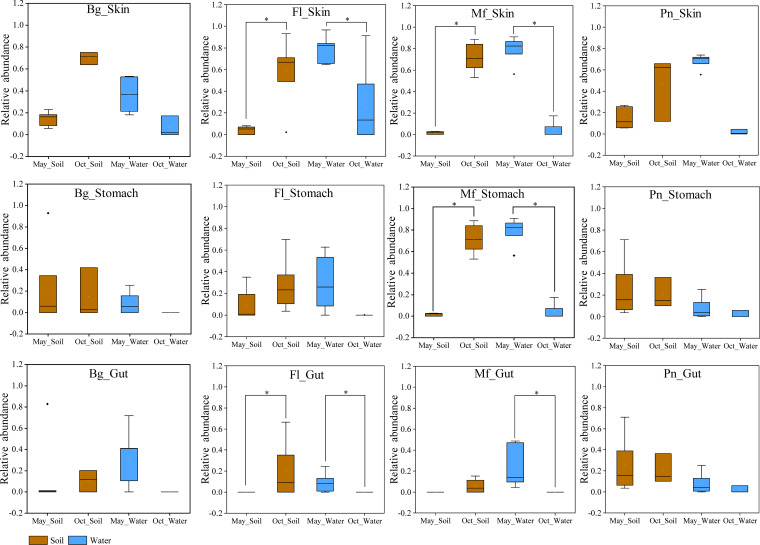
Box plots of the putative contributions (mean relative abundance) from each environmental microbiome to the symbiotic microbiomes of each frog species in May and October (Oct). The Mann-Whitney *U* test was used to analyze the differences between the May and October samples in each type of symbiotic microbiome within the same frog species. *, *P* < 0.002 after the Dunn-Sidàk correction. Bg, *Bufo gargarizans* samples; Fl, *Fejervarya limnocharis* samples; Mf, *Microhyla fissipes* samples; Pn, *Pelophylax nigromaculatus* samples. Blue and brown indicate water source and soil source microbiomes, respectively. Each row shows the analyses based on one kind of the symbiotic microbiome. The upper and lower whiskers represent scores outside the middle 50% (i.e., the lower 25% of scores and the upper 25% of scores). The minimum score is the lowest score, excluding outliers (shown at the end of the down whisker). The maximum score is the highest score, excluding outliers (shown at the end of the top whisker). In the boxes, the upper lines represent the upper quartiles (75th percentiles), the bottom lines represent the lower quartiles (25th percentiles), the lines between the upper and bottom lines represent the median values, and the squares represent mean values. The black diamonds represent the outliers.

The putative contributions of the water source microbiome to each type of symbiotic microbiomes in the frogs were higher in samples obtained in May than in those in October, especially in F. limnocharis and M. fissipes (Fig. 4 and 5). The putative contributions of the soil source microbiome to frog symbiotic microbiomes, particularly the skin and stomach microbiomes, were higher in samples obtained in October than in those obtained in May, especially in F. limnocharis and M. fissipes (Fig. 4 and 5).

Furthermore, we calculated the main putative transmission microbiome from the living environment and found consensus patterns across four frog species. For example, in May (Table 1), the main consensus transmission microbes from the soil source included operational taxonomic unit 2 (OTU2), OTU26, and OTU44 in skin samples, OTU1, OTU5, OTU6, OTU17, and OTU26 in stomach samples, and OTU17, OTU18, and OTU42 in gut samples. In October samples (Table 1), the main consensus transmission microbes from the soil source included OTU1, OTU2, OTU8, OTU11, OTU14, OTU19, OTU105, (unclassified), and OTU117 in skin samples, OTU1, OTU2, OTU8, and OTU27 in stomach samples, and OTU1 in gut samples.

**TABLE 1:** Contribution of the soil source microbiome (showing the top 10 OTUs) to the symbiotic microbiome of each frog species^*a*^

Species	Skin sample		Stomach sample		Gut sample	
	May	October	May	October	May	October
*Bufo gargarizans*	**OTU26 (g__*Carnobacterium*)**	**OTU105 (unclassified)**	**OTU6 (g__*Lactococcus*)**	**OTU1 (g__*Enterobacter*)**	**OTU17 (g__*Bacillus*)**	**OTU1 (g__*Enterobacter*)**
	OTU124 (g__*Lysobacter*)	**OTU8 (g__*Klebsiella*)**	**OTU1 (g__*Enterobacter*)**	**OTU27 (g__*Enterobacter*)**	**OTU18 (g__*Lactococcus*)**	OTU24 (g__*Paraclostridium*)
	**OTU2 (g__** **Acinetobacter)**	**OTU19 (g__*Propionibacterium*)**	OTU18 (g__*Lactococcus*)	OTU252 (c__chloroplast)	OTU5 (g__*Sphingobium*)	OTU99 (g__*Bacteroides*)
	OTU89 (g__*Microbacterium*)	**OTU14 (g__*Curvibacter*)**	**OTU17 (g__*Bacillus*)**	**OTU8 (g__*Klebsiella*)**	OTU1 (g__*Enterobacter*)	OTU8 (g__*Klebsiella*)
	**OTU44 (g__*Pseudarthrobacter*)**	**OTU2 (g__** **Acinetobacter)**	OTU4 (o__*Clostridiales*)	OTU51 (g__*Buttiauxella*)	OTU26 (g__*Carnobacterium*)	OTU7 (g__*Aeromonas*)
	OTU1 (g__*Enterobacter*)	**OTU1 (g__*Enterobacter*)**	**OTU26 (g__*Carnobacterium*)**	OTU7 (g__*Aeromonas*)	OTU6 (g__*Lactococcus*)	OTU19 (g__*Propionibacterium*)
	OTU91 (g__*Nocardioides*)	OTU164 (g__*Lactobacillus*)	**OTU5 (g__*Sphingobium*)**	OTU42 (g__*Enterococcus*)	**OTU42 (g__*Enterococcus*)**	OTU22 (c__chloroplast)
	OTU84 (g__*Pseudomonas*)	**OTU117 (g__*Lactobacillus*)**	OTU118 (g__*Bacillus*)	OTU166 (c__chloroplast)	OTU118 (g__*Bacillus*)	OTU47 (g__*Parabacteroides*)
	OTU300 (g__*Tessaracoccus*)	OTU287 (f__*Ruminococcaceae*)	OTU15 (g__*Exiguobacterium*)	OTU279 (f__mitochondria)	OTU298 (g__*Streptococcus*)	OTU350 (g__*Lelliottia*)
	OTU135 (g__*Bacillus*)	OTU22 (c__chloroplast)	OTU134 (g__*Sphingopyxis*)	OTU12 (g__*Sphingomonas*)	OTU4 (o__*Clostridiales*)	OTU444 (unclassified)
*Fejervarya limnocharis*	**OTU26 (g__*Carnobacterium*)**	**OTU19 (g__*Propionibacterium*)**	**OTU6 (g__*Lactococcus*)**	**OTU1 (g__*Enterobacter*)**	**OTU17 (g__*Bacillus*)**	**OTU1 (g__*Enterobacter*)**
	**OTU44 (g__*Pseudarthrobacter*)**	OTU22 (c__chloroplast)	**OTU5 (g__*Sphingobium*)**	**OTU27 (g__*Enterobacter*)**	**OTU18 (g__*Lactococcus*)**	OTU8 (g__*Klebsiella*)
	OTU166 (c__chloroplast)	**OTU2 (g__** **Acinetobacter)**	**OTU1 (g__*Enterobacter*)**	**OTU8 (g__*Klebsiella*)**	OTU118 (g__*Bacillus*)	OTU7 (g__*Aeromonas*)
	OTU365 (f__*Bradyrhizobiaceae*)	**OTU8 (g__*Klebsiella*)**	**OTU26 (g__*Carnobacterium*)**	OTU7 (g__*Aeromonas*)	OTU14 (g__*Curvibacter*)	OTU27 (g__*Enterobacter*)
	OTU312 (g__*Brevundimonas*)	**OTU1 (g__*Enterobacter*)**	OTU134 (g__*Sphingopyxis*)	OTU2 (g__Acinetobacter)	**OTU42 (g__*Enterococcus*)**	OTU24 (g__*Paraclostridium*)
	OTU38 (g__*Rhizobium*)	**OTU14 (g__*Curvibacter*)**	OTU70 (g__*Afipia*)	OTU329 (o__*Clostridiales*)	OTU11 (g__*Bacteroides*)	OTU29 (g__*Bacteroides*)
	OTU84 (g__*Pseudomonas*)	OTU144 (g__*Methylobacterium*)	OTU109 (g__*Massilia*)	OTU24 (g__*Paraclostridium*)	OTU298 (g__*Streptococcus*)	OTU227 (g__*Epulopiscium*)
	**OTU2 (g__** **Acinetobacter)**	OTU7 (g__*Aeromonas*)	OTU151 (g__*DomiBacillus*)	OTU367 (g__*Acidibacter*)	OTU166 (c__chloroplast)	OTU212 (g__*Intestinibacter*)
	OTU156 (g__*Stenotrophomonas*)	OTU127 (g__*Pseudomonas*)	OTU8 (g__*Klebsiella*)	OTU771 (g__*Xanthomonas*)	—^*b*^	OTU2 (g__Acinetobacter)
	OTU423 (g__*Stenotrophomonas*)	**OTU117 (g__*Lactobacillus*)**	**OTU17 (g__*Bacillus*)**	OTU215 (p__*Chloroflexi*)	—	OTU135 (g__*Bacillus*)
*Microhyla fissipes*	**OTU44 (g__*Pseudarthrobacter*)**	**OTU2 (g__** **Acinetobacter)**	**OTU6 (g__*Lactococcus*)**	**OTU8 (g__*Klebsiella*)**	—	**OTU1 (g__*Enterobacter*)**
	**OTU26 (g__*Carnobacterium*)**	**OTU19 (g__*Propionibacterium*)**	**OTU1 (g__*Enterobacter*)**	**OTU1 (g__*Enterobacter*)**	—	OTU8 (g__*Klebsiella*)
	OTU18 (g__*Lactococcus*)	**OTU8 (g__*Klebsiella*)**	OTU44 (g__*Pseudarthrobacter*)	OTU164 (g__*Lactobacillus*)	—	OTU11 (g__*Bacteroides*)
	OTU17 (g__*Bacillus*)	**OTU1 (g__*Enterobacter*)**	OTU84 (g__*Pseudomonas*)	OTU183 (g__*Lactobacillus*)	—	OTU22 (c__chloroplast)
	OTU3 (f__*Caulobacteraceae*)	OTU114 (o__*Bacteroidales*)	OTU135 (g__*Bacillus*)	OTU262 (o__*Bacteroidales*)	—	OTU135 (g__*Bacillus*)
	OTU430 (g__*Leuconostoc*)	OTU81 (o__*Bacteroidales*)	**OTU26 (g__*Carnobacterium*)**	OTU254 (g__*Lactobacillus*)	—	OTU302 (g__*Bacillus*)
	OTU165 (g__*Bacillus*)	**OTU14 (g__*Curvibacter*)**	OTU2 (g__Acinetobacter)	OTU149 (g__*Faecalibaculum*)	—	OTU117 (g__*Lactobacillus*)
	**OTU2 (g__** **Acinetobacter)**	OTU155 (g__*Rhizobium*)	**OTU17 (g__*Bacillus*)**	**OTU27 (g__*Enterobacter*)**	—	OTU186 (g__*Bacteroides*)
	OTU135 (g__*Bacillus*)	**OTU105 (unclassified)**	OTU246 (g__*Pseudomonas*)	OTU22 (c__chloroplast)	—	OTU33 (g__*Parabacteroides*)
	OTU54 (g__*Pseudoalteromonas*)	OTU280 (f__*Lachnospiraceae*)	OTU750 (g__*Cupriavidus*)	OTU278 (g__*Lactobacillus*)	—	OTU678 (g__*Roseburia*)
*Pelophylax* *nigromaculatus*	OTU5 (g__*Sphingobium*)	**OTU2 (g__** **Acinetobacter)**	**OTU6 (g__*Lactococcus*)**	**OTU1 (g__*Enterobacter*)**	**OTU18 (g__*Lactococcus*)**	**OTU1 (g__*Enterobacter*)**
	**OTU26 (g__*Carnobacterium*)**	**OTU8 (g__*Klebsiella*)**	**OTU1 (g__*Enterobacter*)**	OTU149 (g__*Faecalibaculum*)	**OTU17 (g__*Bacillus*)**	OTU27 (g__*Enterobacter*)
	OTU134 (g__*Sphingopyxis*)	**OTU105 (unclassified)**	**OTU5 (g__*Sphingobium*)**	**OTU8 (g__*Klebsiella*)**	OTU1 (g__*Enterobacter*)	OTU2 (g__Acinetobacter)
	**OTU44 (g__*Pseudarthrobacter*)**	**OTU1 (g__*Enterobacter*)**	OTU18 (g__*Lactococcus*)	OTU164 (g__*Lactobacillus*)	OTU16 (o__*Clostridiales*)	OTU7 (g__*Aeromonas*)
	OTU1 (g__*Enterobacter*)	OTU349 (g__*Leifsonia*)	OTU16 (o__*Clostridiales*)	OTU81 (o__*Bacteroidales*)	OTU6 (g__*Lactococcus*)	OTU8 (g__*Klebsiella*)
	OTU70 (g__Acinetobacter)	**OTU117 (g__*Lactobacillus*)**	**OTU17 (g__*Bacillus*)**	OTU309 (o__*Bacteroidales*)	OTU26 (g__*Carnobacterium*)	OTU16 (o__*Clostridiales*)
	**OTU2 (g__** **Acinetobacter)**	OTU81 (o__*Bacteroidales*)	**OTU26 (g__*Carnobacterium*)**	OTU262 (o__*Bacteroidales*)	OTU118 (g__*Bacillus*)	OTU11 (g__*Bacteroides*)
	OTU118 (g__*Bacillus*)	OTU287 (f__*Ruminococcaceae*)	OTU2 (g__Acinetobacter)	OTU51 (g__*Buttiauxella*)	**OTU42 (g__*Enterococcus*)**	OTU99 (g__*Bacteroides*)
	OTU151 (g__*Domibacillus*)	OTU155 (g__*Rhizobium*)	OTU165 (g__*Bacillus*)	OTU22 (c__chloroplast)	OTU8 (g__*Klebsiella*)	OTU108 (f__*Lachnospiraceae*)
	OTU139 (g__*Aerococcus*)	OTU164 (g__*Lactobacillus*)	OTU118 (g__*Bacillus*)	OTU183 (g__*Lactobacillus*)	OTU430 (g__*Leuconostoc*)	OTU47 (g__*Parabacteroides*)

a Bold font indicates the consensus pattern, i.e., the OTU appeared in the microbiome of three or more frog species within each group. Soil source microbiome in the host symbiotic microbiome meant that the host likely acquired the microbiome from the land environment.

b —, no contribution.

In May samples (Table 2), the main consensus transmission microbes from the water source included OTU1, OTU2, OTU3, OTU7, and OTU31 in skin samples, OTU1, OTU2, OTU3, OTU5, OTU7, OTU12, and OTU15 in stomach samples, and OTU1, OTU7, OTU8, OTU9, and OTU13 in gut samples. In October (Table 2), the main consensus transmission microbes from the water source included OTU2, OTU14, and OTU71 in skin samples. The contributions of the water microbiome to the stomach and gut microbiomes were low in October.

**TABLE 2:** Contribution of the water source microbiome (showing the top 10 OTUs) to the symbiotic microbiome of each frog species^*a*^

Species	Skin sample		Stomach sample		Gut sample	
	May	October	May	October	May	October
*Bufo gargarizans*	**OTU2 (g__** **Acinetobacter)**	**OTU14 (g__*Curvibacter*)**	**OTU5 (g__*Sphingobium*)**	—^*b*^	**OTU1 (g__*Enterobacter*)**	—
	**OTU1 (g__*Enterobacter*)**	**OTU2 (g__** **Acinetobacter)**	**OTU1 (g__*Enterobacter*)**	—	**OTU9 (g__*Parabacteroides*)**	—
	**OTU7 (g__*Aeromonas*)**	OTU176 (g__*Methylotenera*)	**OTU7 (g__*Aeromonas*)**	—	OTU25 (g__*Bacteroides*)	—
	OTU89 (g__*Microbacterium*)	OTU287 (f__*Ruminococcaceae*)	**OTU15 (g__*Exiguobacterium*)**	—	OTU5 (g__*Sphingobium*)	—
	OTU15 (g__*Exiguobacterium*)	OTU339 (g__*Sediminibacterium*)	**OTU12 (g__*Sphingomonas*)**	—	**OTU7 (g__*Aeromonas*)**	—
	OTU242 (g__*Agrococcus*)	OTU352 (f__*Ruminococcaceae*)	**OTU3 (f__*Caulobacteraceae*)**	—	**OTU13 (g__*Bacteroides*)**	—
	OTU76 (g__*Desemzia*)	**OTU71 (f__*Comamonadaceae*)**	**OTU2 (g__** **Acinetobacter)**	—	OTU24 (g__*Paraclostridium*)	—
	OTU26 (g__*Carnobacterium*)	OTU32 (g__Acinetobacter)	OTU14 (g__*Curvibacter*)	—	**OTU8 (g__*Klebsiella*)**	—
	**OTU31 (g__*Ensifer*)**	OTU847 (f__*Christensenellaceae*)	OTU43 (g__*Caulobacter*)	—	OTU51 (g__*Buttiauxella*)	—
	OTU32 (g__Acinetobacter)	OTU545 (g__*Zoogloea*)	OTU24 (g__*Paraclostridium*)	—	OTU192 (f__*Ruminococcaceae*)	—
*Fejervarya limnocharis*	**OTU3 (f__*Caulobacteraceae*)**	**OTU14 (g__*Curvibacter*)**	**OTU1 (g__*Enterobacter*)**	OTU90 (g__*Flavobacterium*)	**OTU1 (g__*Enterobacter*)**	—
	OTU15 (g__*Exiguobacterium*)	**OTU2 (g__** **Acinetobacter)**	**OTU3 (f__*Caulobacteraceae*)**	OTU634 (g__*Fluviicola*)	**OTU9 (g__*Parabacteroides*)**	—
	**OTU2 (g__** **Acinetobacter)**	OTU45 (f__*Prevotellaceae*)	**OTU5 (g__*Sphingobium*)**	OTU340 (g__*Faecalibacterium*)	**OTU7 (g__*Aeromonas*)**	—
	**OTU12 (g__*Sphingomonas*)**	OTU7 (g__*Aeromonas*)	**OTU7 (g__*Aeromonas*)**	OTU85 (g__*Aquabacterium*)	**OTU8 (g__*Klebsiella*)**	—
	**OTU31 (g__*Ensifer*)**	OTU326 (f__*Prevotellaceae*)	**OTU2 (g__** **Acinetobacter)**	OTU120 (g__*Rheinheimera*)	OTU41 (f__*Rikenellaceae*)	—
	**OTU1 (g__*Enterobacter*)**	OTU28 (g__*Comamonas*)	**OTU15 (g__*Exiguobacterium*)**	OTU339 (g__*Sediminibacterium*)	OTU2 (g__Acinetobacter)	—
	OTU38 (g__*Rhizobium*)	OTU85 (g__*Aquabacterium*)	OTU28 (g__*Comamonas*)	OTU712 (g__*Sphingopyxis*)	OTU23 (g__*Parabacteroides*)	—
	**OTU7 (g__*Aeromonas*)**	OTU207 (g__*Arcobacter*)	OTU4 (o__*Clostridiales*)	OTU995 (c__chloroplast)	OTU25 (g__*Bacteroides*)	—
	OTU28 (g__*Comamonas*)	**OTU71 (f__*Comamonadaceae*)**	OTU109 (g__*Massilia*)	OTU1020 (f__*Hydrogenophilaceae*)	**OTU13 (g__*Bacteroides*)**	—
	OTU67 (g__*Maritimibacter*)	OTU516 (g__*Tolumonas*)	**OTU12 (g__*Sphingomonas*)**	OTU2121 (g__*Flavobacterium*)	OTU39 (g__*Parabacteroides*)	—
*Microhyla fissipes*	**OTU3 (f__*Caulobacteraceae*)**	**OTU14 (g__*Curvibacter*)**	**OTU3 (f__*Caulobacteraceae*)**	OTU244 (f__*Rhodocyclaceae*)	**OTU1 (g__*Enterobacter*)**	—
	**OTU2 (g__** **Acinetobacter)**	OTU287 (f__*Ruminococcaceae*)	**OTU1 (g__*Enterobacter*)**	OTU28 (g__*Comamonas*)	OTU39 (g__*Parabacteroides*)	—
	**OTU12 (g__*Sphingomonas*)**	**OTU2 (g__** **Acinetobacter)**	**OTU2 (g__** **Acinetobacter)**	OTU545 (g__*Zoogloea*)	**OTU13 (g__*Bacteroides*)**	—
	OTU15 (g__*Exiguobacterium*)	OTU352 (f__*Ruminococcaceae*)	**OTU12 (g__*Sphingomonas*)**	OTU25 (g__*Bacteroides*)	OTU35 (p__*Bacteroidetes*)	—
	OTU43 (g__*Caulobacter*)	OTU508 (g__*Vibrio*)	**OTU7 (g__*Aeromonas*)**	OTU186 (g__*Bacteroides*)	**OTU9 (g__*Parabacteroides*)**	—
	**OTU1 (g__*Enterobacter*)**	**OTU71 (f__*Comamonadaceae*)**	OTU32 (g__*Aeromonas*)	OTU1534 (f__*Prevotellaceae*)	OTU62 (g__*Phascolarctobacterium*)	—
	OTU42 (g__*Enterococcus*)	OTU69 (g__*Sphaerotilus*)	OTU8 (g__*Klebsiella*)	OTU142 (g__*Polynucleobacter*)	**OTU7 (g__*Aeromonas*)**	—
	OTU161 (g__*Flavobacterium*)	OTU804 (f__*Ruminococcaceae*)	**OTU15 (g__*Exiguobacterium*)**	OTU326 (f__*Prevotellaceae*)	OTU3 (f__*Caulobacteraceae*)	—
	**OTU31 (g__*Ensifer*)**	OTU188 (g__*Brevundimonas*)	OTU54 (g__*Pseudoalteromonas*)	OTU62 (g__*Phascolarctobacterium*)	OTU56 (g__*Bacteroides*)	—
	OTU54 (g__*Pseudoalteromonas*)	OTU594 (g__*Phascolarctobacterium*)	OTU83 (g__*Streptococcus*)	OTU340 (g__*Faecalibacterium*)	OTU132 (g__*Bacteroides*)	—
*Pelophylax* *nigromaculatus*	OTU5 (g__*Sphingobium*)	OTU203 (g__*Cloacibacterium*)	OTU21 (g__*Cetobacterium*)	OTU7 (g__*Aeromonas*)	**OTU1 (g__*Enterobacter*)**	OTU21 (g__*Cetobacterium*)
	**OTU3 (f__*Caulobacteraceae*)**	OTU349 (g__*Leifsonia*)	**OTU1 (g__*Enterobacter*)**	OTU14 (g__*Curvibacter*)	OTU21 (g__*Cetobacterium*)	OTU1 (g__*Enterobacter*)
	**OTU2 (g__** **Acinetobacter)**	OTU871 (g__*Cloacibacterium*)	OTU2 (g__Acinetobacter)	OTU28 (g__*Comamonas*)	**OTU7 (g__*Aeromonas*)**	OTU25 (g__*Bacteroides*)
	**OTU1 (g__*Enterobacter*)**	OTU61 (g__*Flavobacterium*)	OTU6 (g__*Lactococcus*)	OTU69 (g__*Sphaerotilus*)	**OTU13 (g__*Bacteroides*)**	OTU7 (g__*Aeromonas*)
	OTU28 (g__*Comamonas*)	OTU352 (f__*Ruminococcaceae*)	OTU24 (g__*Paraclostridium*)	OTU21 (g__*Cetobacterium*)	OTU175 (g__*Rickettsiella*)	OTU12 (g__*Alistipes*)
	**OTU7 (g__*Aeromonas*)**	**OTU71 (f__*Comamonadaceae*)**	**OTU7 (g__*Aeromonas*)**	OTU1 (g__*Enterobacter*)	OTU40 (g__*Rickettsiella*)	OTU6 (g__*Lactococcus*)
	**OTU12 (g__*Sphingomonas*)**	OTU287 (f__*Ruminococcaceae*)	OTU8 (g__*Klebsiella*)	OTU863 (g__*Thauera*)	OTU30 (g__*Rickettsiella*)	OTU8 (g__*Klebsiella*)
	OTU32 (g__Acinetobacter)	OTU86 (g__*Limnohabitans*)	OTU13 (g__*Bacteroides*)	OTU1371 (f__*Prevotellaceae*)	**OTU9 (g__*Parabacteroides*)**	OTU1252 (g__*Cetobacterium*)
	OTU8 (g__*Klebsiella*)	OTU188 (g__*Brevundimonas*)	OTU28 (g__*Comamonas*)	OTU902 (g__*Acidithiobacillus*)	OTU27 (g__*Enterobacter*)	OTU186 (g__*Bacteroides*)
	OTU43 (g__*Caulobacter*)	**OTU2 (g__** **Acinetobacter)**	**OTU5 (g__*Sphingobium*)**	OTU52 (g__*Hydrogenophaga*)	**OTU8 (g__*Klebsiella*)**	OTU193 (f__FamilyXIII)

a Bold font indicates the consensus pattern, i.e., the OTU appeared in the microbiome of three or more frog species within each group. Water source microbiome in the host symbiotic microbiome meant that the host likely acquired the microbiome from the aquatic environment.

b —, no contribution.

In this study, we also investigated the species differences in the microbiome transmission among these four species. Here, we found that B. gargarizans harbored the lowest proportion of putative water source microbiome (∼36%) in the skin microbiome compared to that in the other three species in May samples (F. limnocharis, ∼79%; M. fissipes, ∼77%; P. nigromaculatus, ∼69%) (Fig. 5). P. nigromaculatus harbored the lowest proportion of putative soil source microbiome (∼46%) in skin microbiome compared to that in other three species in October samples (B. gargarizans, ∼70%; F. limnocharis, ∼73%; M. fissipes, ∼72%) (Fig. 5).

### Potentially Batrachochytrium dendrobatidis-inhibitory bacteria in May and October.

**FIG 6:**
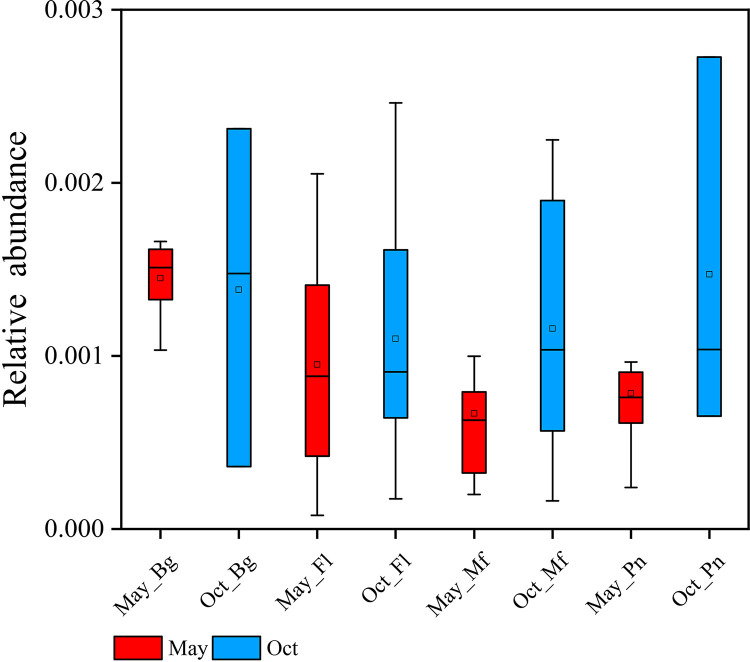
Mean relative abundances of the potentially *Batrachochytrium dendrobatidis*-inhibitory bacteria in the skin samples in the four frog species. The Mann-Whitney *U* test was used to analyze the differences between samples obtained in May and October in the skin microbiome within the same frog species (all *P* > 0.05). Bg, *Bufo gargarizans* samples; Fl, *Fejervarya limnocharis* samples; Mf, *Microhyla fissipes* samples; Pn, *Pelophylax nigromaculatus* samples. The upper and lower whiskers represent scores outside the middle 50% (i.e., the lower 25% of scores and the upper 25% of scores). The minimum score is the lowest score, excluding outliers (shown at the end of the down whisker). The maximum score is the highest score, excluding outliers (shown at the end of the top whisker). In the boxes, the upper lines represent the upper quartiles (75th percentiles), the bottom lines represent the lower quartiles (25th percentiles), the lines between the upper and bottom lines represent the median values, and the squares represented mean values. The black diamonds represent the outliers.

Finally, we evaluated the abundance of putative B. dendrobatidis-inhibitory bacteria in the skin samples from each frog species and found no significant difference between May and October (Fig. 6) (P > 0.05).

## DISCUSSION

Host diet and phylogeny are the two main factors influencing gut microbiomes (29, 30). Symbiotic microbiome communities vary across the body (31). Our previous studies revealed the differences in the skin, stomach, and gut microbiomes in amphibians (32, 33). Here, significant differences in the symbiotic microbiome communities on the skin and in the stomach and gut within each species (occupying site factor) were observed.

### Influence of the environmental microbiome on temporal dynamics of these frogs’ symbiotic microbiomes.

Seasonal changes in the skin microbiome have been reported in several vertebrate species, including amphibians (8, 34, 35) and whales (36). Environmental factors, such as water physical and chemical properties, and potential pathogens may influence the skin microbiome. Studies revealed that the seasonal dynamics in the animal gut microbiome might be caused by dietary changes (37–40). We speculated that studies investigating the seasonal changes in the symbiotic microbiome in amphibians might neglect the effect of the host behavior. We uncovered the consistent directional changes in the relative of soil source and water source microbiomes over time for these four species. The results revealed that the frogs harbored more water source microbes in their symbiotic microbiome in May than in October. On the contrary, the frogs harbored more soil source microbes in their symbiotic microbiome in October than in May. The four frog species investigated prefer to live in a water environment in May. However, as the environmental temperature decreases in October, the frogs prefer to live on land in preparation for hibernation in caves or under stones (23–25). Thus, the seasonal changes in the symbiotic microbiomes in amphibians may be caused by the difference in the microbiome transmission from their living environment due to their behavior. Here, we brought a simple example of how the behavior and living environment of wild amphibians shape their symbiotic microbiome. This finding increased the understanding of the interactions between host behavior and microbiome dynamics in the field of microbial ecology.

Furthermore, in the skin microbiome, we found that the host species differences in the microbiome transmission in this study, which might be caused by the strength of the relationship between environment and microbiome, vary in concert with species due to host behavior. For example, B. gargarizans prefers to select bare surfaces or low vegetable as feeding sites in southwestern China and uses daily shelters similar to those of other terrestrial amphibians (22). Here, the skin microbiome of B. gargarizans harbored the lowest proportion of a putative water source microbiome in the May samples in this study. Therefore, we demonstrated that external (skin) and internal (stomach and gut) symbiotic microbiomes are shaped by amphibian behavior and living environments along with potential species differences. We reported the potential association between specific behaviors in poikilothermic animals and host symbiotic microbiomes.

We also revealed that common transmission microbes included *Proteobacteria*, such as *Enterobacter* and Acinetobacter, which live in natural environments such as soil and water (41, 42). The majority of the main transmission microbes from soil or water were different due to the difference in environments. For example, one of the main putative transmission microbes from the water was *Aeromonas* (*Proteobacteria*), which predominantly lives in a water environment (43, 44). Thus, the difference in the transmission microbes may lead to the differences in the frog species observed between May and October.

### Potential connection between the frog’s symbiotic microbiome and pathogen resistance.

A previous study revealed that winter skin samples of two amphibians (*Lithobates* [*Rana*] *yavapaiensis* and Eleutherodactylus coqui) had high B. dendrobatidis susceptibility and bacterial diversity (34). However, a significant difference in putative B. dendrobatidis susceptibility and bacterial diversity was not reported in the present study, which was based on similar analytical methods. This may be attributed to the different species or habitats investigated. Moreover, the study reveals the European common frog (Rana temporaria) with higher preexposure skin microbiome diversity appears to exhibit higher survival to a lethal viral pathogen, *Ranavirus* (10). In our study, we did not find a significant consensus for changes in the alpha diversity (e.g., Shannon index) between the Many and October sample across four species. Also, we did not perform the pathogen exposure experiments. Thus, we are unable to speculate on the putative relationship between pathogen resistance and the temporal dynamics of the skin microbiome in these four species in this study. Also, we observed temporal changes in the gut microbiome between the Many and October samples in each species in this study. One previous study on host-microbe interactions suggested that the changes in the gut microbiome shape phenotypes across ontogeny in amphibians (e.g., the wood frog Rana sylvatica) (45). Therefore, it will be of interest to reveal in future studies the potential host-microbe interactions in amphibians considering the temporal changes in their symbiotic microbiomes (e.g., skin, stomach, and gut microbiomes) caused by the environmental influence due to their special behaviors.

### Conclusion.

The present study revealed that wild amphibian behavior influences their external and internal symbiotic microbiome communities through changes in their living environment. This suggests that the globally changing environment may influence the development of wild animal symbiotic microbiomes.

## MATERIALS AND METHODS

### Sample collection.

A total of 80 skin (May: 8, B. gargarizans; 18, F. limnocharis; 15, M. fissipes; 12, P. nigromaculatus; October: 3, B. gargarizans; 15, F. limnocharis; 6, M. fissipes; 3, P. nigromaculatus), 65 stomach (May: 15, B. gargarizans; 8, F. limnocharis; 15, M. fissipes; 12, P. nigromaculatus; October: 3, B. gargarizans; 5, F. limnocharis; 4, M. fissipes; 3, P. nigromaculatus) , and 88 gut (May: 15, B. gargarizans; 20, F. limnocharis; 16, M. fissipes; 13, P. nigromaculatus; October: 3 B. gargarizans, 14 F. limnocharis, 4 M. fissipes, and 3 P. nigromaculatus) samples from four wild frog species and 59 environment samples (May: 11 water and 21 soil samples; October: 9 water and 18 soil samples) were collected in the Leshan mountains (Sichuan, China) in May and October in 2018 (Fig. 1; see also Table S1 in the supplemental material). All instruments and materials were sterilized prior to sampling. The frogs were collected using nets. In May, the four frog species were collected from wet rice fields; however, in October, the frogs were collected from dry rice fields and vegetable fields.

For skin microbial sampling, sterile water was used to rinse the frogs three times to remove potential transient bacteria prior to collecting the skin microbes (46). To standardize sample collection, sterile swabs that did not exhibit germicidal effects on the microbes were used to wipe the dorsal, ventral, and lateral sides of the frogs. For gut and stomach microbial sampling, each frog was euthanized and dissected to collect the gut and stomach contents in 2-ml aseptic centrifuge tubes. For environmental sampling, each water sample was collected in two 5-liter sterile polyethylene terephthalate (PET) bottles and immediately stored at −20°C (47). Then, the water samples were filtered using a vacuum pump. The pressure was 0.5 MPa, the membrane aperture was 0.2 μm, and the diameter was 10 cm (48). Each soil sample (2.5 cm in diameter and 13 cm deep) was collected three times used an aseptic shovel from one sampling site (49) and immediately transferred to sterile sealing bags for preservation. All skin, water, soil, gut, and stomach samples were transferred to a −20°C portable refrigerator on the way to the laboratory. Our experiments were approved by the Institution of Animal Care and the Ethics Committee of Chengdu Institute of Biology, Chinese Academy of Sciences (permit no. 2017-AR-JJP-03).

### DNA extraction and sequencing.

The QIAamp DNA Stool minikit (Qiagen, Valencia, CA) was used to extract DNA from the samples at room temperature. The V4 region of the 16S rRNA gene was amplified with 515F (5′-GTGCCAGCMGCCGCGGTAA-3′) and 806R (5′-GACTACHVGGGTWTCTAAT-3′) primers (50). We used the following PCR thermocycling conditions: 95°C for 5 min, 35 cycles of 95°C for 30 s, 55°C for 30 s, and 72°C for 45 s, with a final extension step at 72°C for 10 min. High-throughput sequencing of amplicons was performed using the Illumina MiSeq platform. Sequencing was performed by Mingke Biotechnology Co., Ltd. (Hangzhou, China).

### 16S rRNA gene-based sequence analysis.

QIIME 1.9 was used to process the raw sequences and to obtain clean sequences, as previously described (51). In this trimming analysis, the search function was used for chimerism checks to remove low-quality sequences, the flash function was used for splicing, and the trimmomatic function was used for quality control (52). Operational taxonomic units (OTUs) were defined as sharing >97% sequence identity by annotating clean sequences to the SILVA132 database (53). The taxon summary was conducted using the OTUs table in QIIME 1.9 (51).

### Alpha diversity analysis.

The alpha diversity was calculated using the observed OTU number. The heatplus package (54) in R was used to generate a heat map for the mean abundance of the phylum, family, and genus of the microbes in the frog gut and environmental samples. The differences in microbial compositions between May and October were compared using the linear discriminant analysis (LDA) effect size (LEfSe) method (55). Mann-Whitney *U* tests were used to investigate the differences in the microbial alpha diversity in each type of symbiotic microbiome between the May and October samples within each species. Within each type of the symbiotic microbiome, there were the samples from May and October among four species. Thus, there were in total eight groups in the multiple testing. We select the Dunn-Sidàk correction to make the strict and conservative *P* value correction (56). After correction, the new significant *P* value was approximately 0.002, and the real *P* value in the Mann-Whitney *U* test below this new *P* value was considered significant.

### Beta diversity analysis.

The adonis function in the vegan package (57) performs a PERMANOVA based on dissimilarity matrices using the OTU table (unweighted UniFrac distances) and was used to compute an *R*^2^ value, which showed the percentage of seasonal variation (May and October). Nonmetric multidimensional scaling (NMDS) (58) was used to visualize the dissimilarity.

### Source-tracking analysis for potential microbiome transmission.

Source-Tracker 0.9.5 (59) was used to assess the contribution (microbiome transmission) of the soil source and water source microbiomes in the samples. The difference between water and soil contributions in each sampling season for each frog species was calculated. For the water source microbiome in the host symbiotic microbiome, the host likely acquired the microbiome from the aquatic environment. For the soil source microbiome in the host symbiotic microbiome, the host likely acquired the microbiome from the land environment. The Mann-Whitney *U* test was used to analyze the differences between the May and October samples in each type of symbiotic microbiome within the same frog species. Within each type of the symbiotic microbiome, there were the samples from May and October among four species. Thus, there were in total eight groups in the multiple testing. We selected the Dunn-Sidàk correction to make the strict and conservative *P* value correction (56). After correction, the new significant *P* value was approximately 0.002, and the real *P* value in Mann-Whitney *U* test below this new *P* value was considered significant.

### Putative pathogen analysis in the frog skin samples.

The clean sequences from the frog samples were analyzed against a database containing >1,900 16S rRNA gene sequences from amphibian skin bacteria that have been tested for activity against the pathogen Batrachochytrium dendrobatidis (60). Then, we identified the potentially B. dendrobatidis-inhibitory OTUs and calculated their relative abundance in each frog sample. Box charts were used to visualize the results. The Mann-Whitney *U* test was used to assess the differences in the mean abundance of the potential B. dendrobatidis-inhibitory OTUs in the frog samples between May and October.

### Data availability.

Sequencing data and relevant files have been uploaded to NCBI with the accession number PRJNA613575. In addition, the 16S data of the skin and gut samples collected in May are part of our previous data (PRJNA549036).
