# Supplementary material for: The Behavior of Amphibians Shapes Their Symbiotic Microbiomes
Source: mSystems. 2020 Jul 28;5(4):e00626-20. doi: 10.1128/mSystems.00626-20 (PMC7394361; doi:10.1128/mSystems.00626-20)
Supplement: TABLE S1 [file mSystems.00626-20-st001.docx]

| **Sampling time** | **Location** | **Scientific name** | **Species (n)** | **Altitude (m)** | **Soil (n)** | **Water (n)** |
| --- | --- | --- | --- | --- | --- | --- |
| May | AG | *Pelophylax nigromaculatus* | Pn (4, 4, 4) | 376 | 3 | 0 |
|  |  | *Microhyla fissipes* | Mf (2, 2, 2) |  |  |  |
|  |  | *Fejervarya limnocharis* | Fl (3, 1, 3) |  |  |  |
| May | GS | *Bufo gargarizans* | Bg (2, 4, 4) | 376 | 3 | 2 |
|  |  | *Fejervarya limnocharis* | Fl (3, 2, 3) |  |  |  |
|  |  | *Microhyla fissipes* | Mf (5, 5, 7) |  |  |  |
| May | XD | *Fejervarya limnocharis* | Fl (4, 3, 5) | 446 | 3 | 2 |
|  |  | *Microhyla fissipes* | Mf (3, 3, 3) |  |  |  |
| May | SB | *Bufo gargarizans* | Bg (5, 10, 10) | 403 | 3 | 1 |
|  |  | *Pelophylax nigromaculatus* | Pn (4, 4, 4) |  |  |  |
| May | MD | *Fejervarya limnocharis* | Fl (4, 1, 5) | 395 | 3 | 2 |
|  |  | *Pelophylax nigromaculatus* | Pn (3, 3, 4) |  |  |  |
|  |  | *Microhyla fissipes* | Mf (1, 1, 1) |  |  |  |
| May | TS | *Fejervarya limnocharis* | Fl (3, 0, 3) | 388 | 3 | 2 |
|  |  | *Pelophylax nigromaculatus* | Pn (1, 1, 1) |  |  |  |
| May | XB | *Bufo gargarizans* | Bg (1, 1, 1) | 360 | 3 | 2 |
|  |  | *Fejervarya limnocharis* | Fl (1, 1, 1) |  |  |  |
|  |  | *Microhyla fissipes* | Mf (4, 4, 3) |  |  |  |
| October | AG | *Pelophylax nigromaculatus* | Pn (3, 3, 3) | 376 | 3 | 2 |
|  |  | *Fejervarya limnocharis* | Fl (3, 1, 3) |  |  |  |
| October | GS | *Fejervarya limnocharis* | Fl (3, 1, 3) | 376 | 3 | 2 |
|  |  | *Microhyla fissipes* | Mf (3, 2, 2) |  |  |  |
| October | XD | *Fejervarya limnocharis* | Fl (3, 1, 3) | 446 | 3 | 0 |
| October | SB | *Bufo gargarizans* | Bg (3, 3, 2) | 403 | 3 | 2 |
| October | MD | *Fejervarya limnocharis* | Fl (3, 1, 3) | 395 | 3 | 1 |
|  |  | *Microhyla fissipes* | Mf (3, 2, 2) |  |  |  |
| October | TS | *Fejervarya limnocharis* | Fl (3, 1, 2) | 388 | 3 | 2 |
| October | XB | *Bufo gargarizans* | Bg (0, 0, 1) | 360 | 0 | 0 |
