# Supplementary figures and images for: The Behavior of Amphibians Shapes Their Symbiotic Microbiomes
Source: mSystems. 2020 Jul 28;5(4):e00626-20. doi: 10.1128/mSystems.00626-20 (PMC7394361; doi:10.1128/mSystems.00626-20)

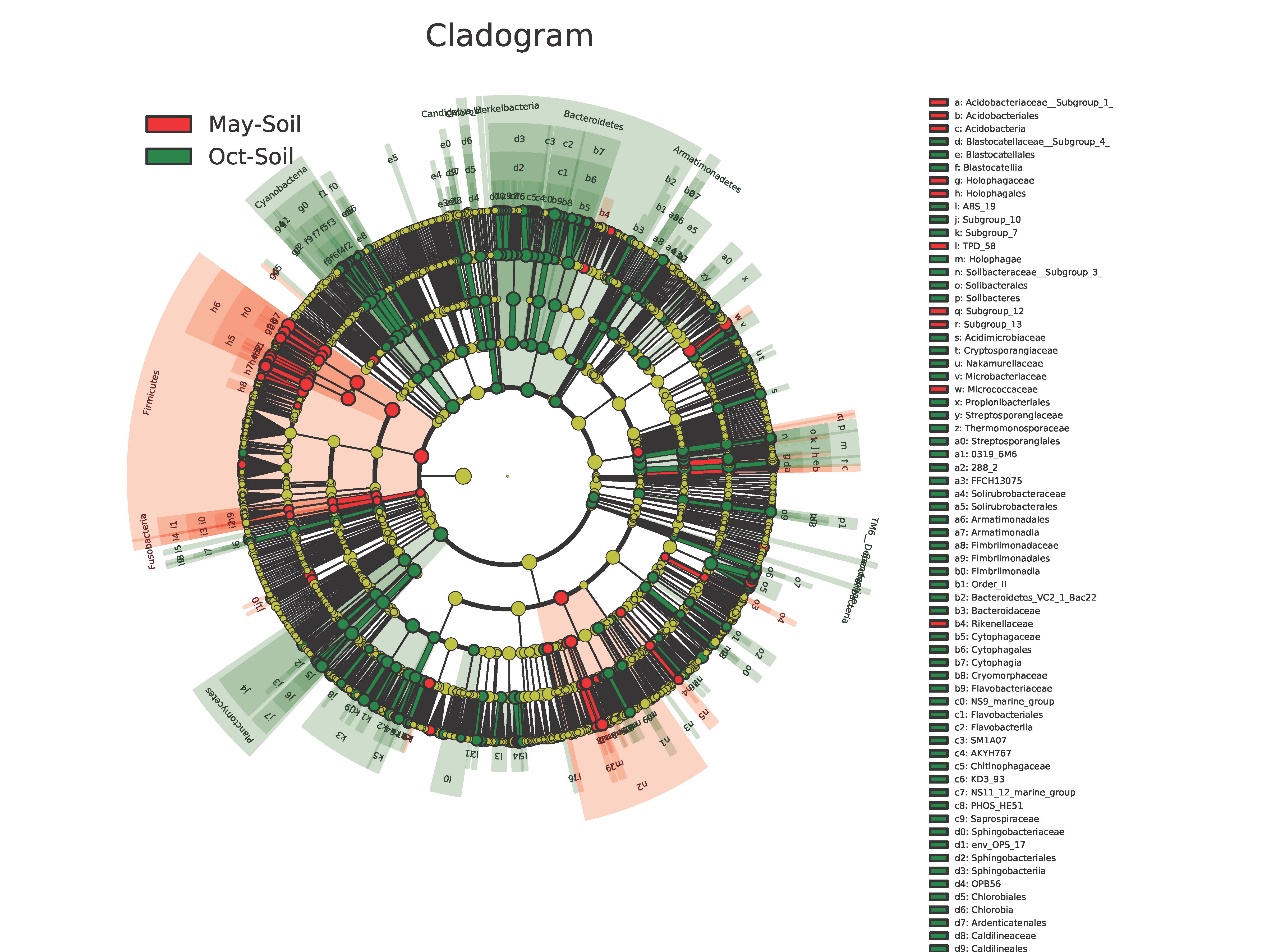

Supplement: FIG S1 [file mSystems.00626-20-sf001.docx]

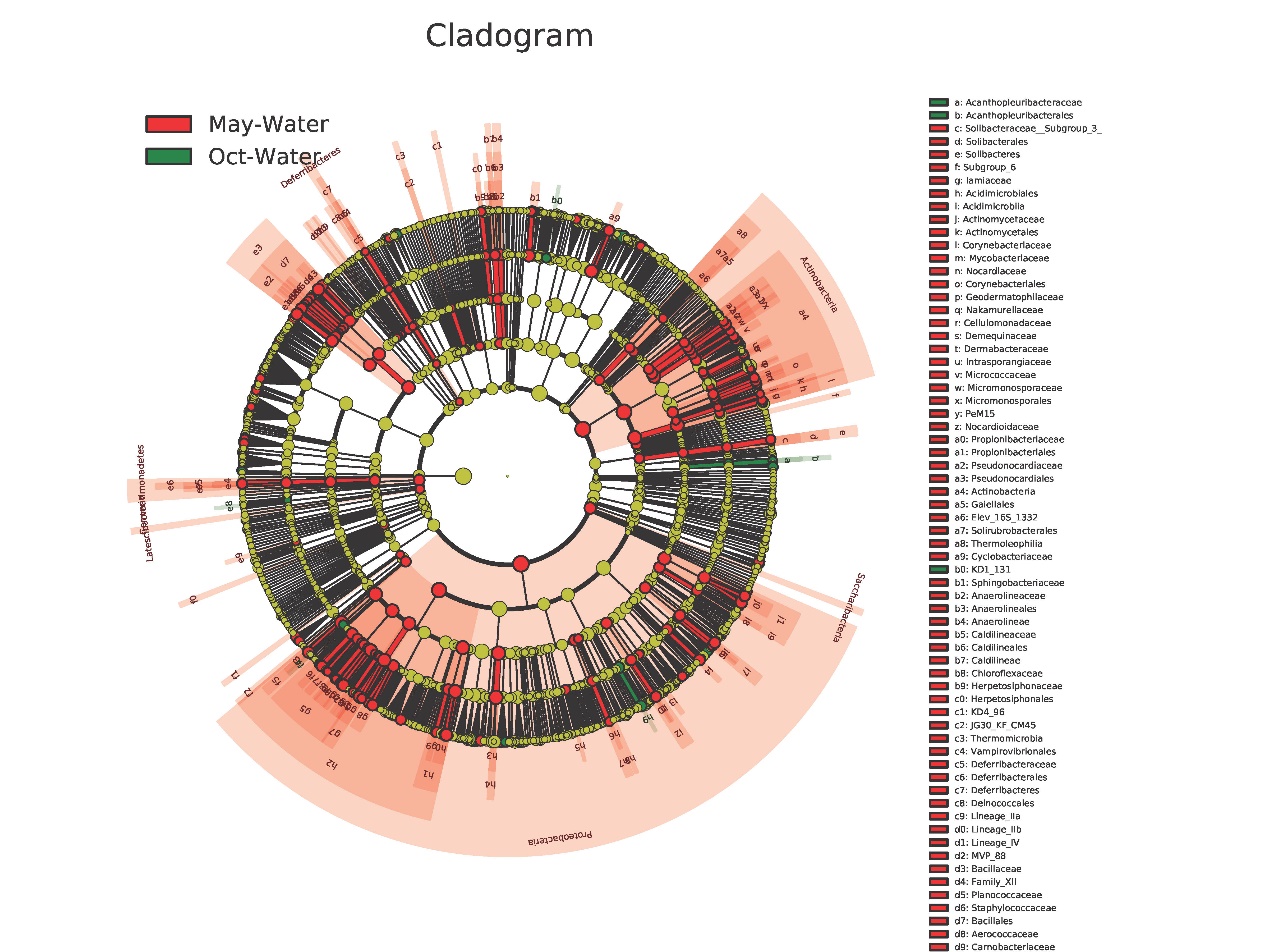

Supplement: FIG S2 [file mSystems.00626-20-sf002.docx]

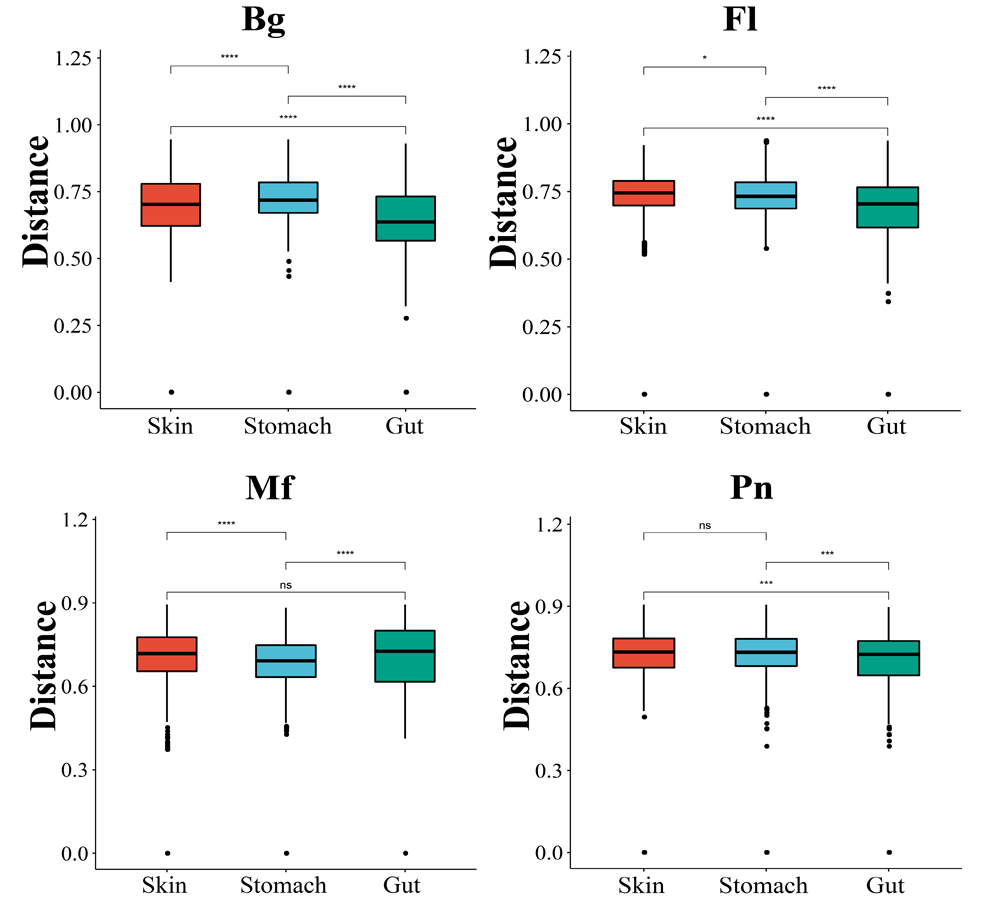

Supplement: FIG S3 [file mSystems.00626-20-sf003.docx]
