# Supplementary material for: The Behavior of Amphibians Shapes Their Symbiotic Microbiomes
Source: mSystems. 2020 Jul 28;5(4):e00626-20. doi: 10.1128/mSystems.00626-20 (PMC7394361; doi:10.1128/mSystems.00626-20)
Supplement: TABLE S2 [file mSystems.00626-20-st002.docx]

|  |  | **Skin** |  | **Stomach** | |  | **Gut** |  | |
| --- | --- | --- | --- | --- | --- | --- | --- | --- | --- |
| **Frog species** |  | **Phylum** | **Family** | **Phylum** | **Family** | | **Phylum** | | **Family** |
| *Bufo*  *gargarizans*  *(Bg)* | May |  | Dermabacteraceae | _ | ***Clostridiaceae_1*** | | _ | Porphyromonadaceae | |
|  |  |  | Micrococcaceae |  | Sphingomonadaceae | |  | Rikenellaceae | |
|  |  |  | Flavobacteriaceae |  | Caulobacteraceae | |  | Bacillaceae | |
|  |  | _ | ***Sphingomonadaceae*** |  |  | |  | Ruminococcaceae | |
|  |  |  | Aeromonadaceae |  |  | |  | Enterobacteriaceae | |
|  |  |  | Moraxellaceae |  |  | |  | Coxiellaceae | |
|  |  |  | Xanthomonadaceae |  |  | |  |  | |
|  | Oct | ***Actinobacteria*** | Erysipelotrichaceae | _ | Spiroplasmataceae | | _ | Bacteroidaceae | |
|  |  |  | Comamonadaceae |  |  | |  |  | |
|  |  |  | Propionibacteriaceae |  |  | |  |  | |
|  |  |  | Lactobacillaceae |  |  | |  |  | |
|  |  |  | Bacteroidales_S24_7_group |  |  | |  |  | |
|  |  |  | Lachnospiraceae |  |  | |  |  | |
| *Fejervarya limnocharis*  *(Fl)* | May | _ | Family_XII | _ | Streptococcaceae | | _ | Bacteroidaceae | |
|  |  |  | Caulobacteraceae |  | ***Clostridiaceae_1*** | |  | Porphyromonadaceae | |
|  |  |  | Rhizoiaceae |  | Anaplasmataceae | |  | Lachnospiraceae | |
|  |  |  |  |  | Sphingomonadaceae | |  | Erysipelotrichaceae | |
|  | Oct | ***Actinobacteria*** | Propionibacteriaceae | _ | _ | | _ | Rikenellaceae | |
|  |  |  | Comamonadaceae |  |  |  |  | Ruminococcaceae | |
|  |  |  | Moraxellaceae |  |  |  |  |  | |
| *Pelophylax nigromaculatus*  *(Pn)* | May | _ | Caulobacteraceae | _ | ***Clostridiaceae_1*** | | _ | Enterobacteriaceae | |
|  |  |  | ***Sphingomonadaceae*** |  |  | |  |  | |
|  |  |  | Moraxellaceae |  |  | |  |  | |
|  | Oct | ***Actinobacteria*** | _ | _ | Bacteroidales_S24_7_group | | _ | Bacteroidaceae | |
|  |  |  |  |  | Streptococcaceae | |  | Porphyromonadaceae | |
|  |  |  |  |  |  | |  | Rikenellaceae | |
|  |  |  |  |  |  | |  | Fusobacteriaceae | |
| *Microhyla fissipes*  *(Mf)* | May | _ | Caulobacteraceae | _ | Enterobacteriaceae | | _ | Lachnospiraceae | |
|  |  |  | ***Sphingomonadaceae*** |  |  | |  | Ruminococcaceae | |
|  |  |  | Moraxellaceae |  |  | |  | Erysipelotrichaceae | |
|  | Oct | ***Actinobacteria*** | Propionibacteriaceae | _ | Lactobacillaceae | | _ | Bacteroidaceae | |
|  |  |  | Bacteroidales_S24_7_group |  |  | |  | Porphyromonadaceae | |
|  |  |  | Comamonadaceae |  |  | |  |  | |
