# Supplementary material for: The Behavior of Amphibians Shapes Their Symbiotic Microbiomes
Source: mSystems. 2020 Jul 28;5(4):e00626-20. doi: 10.1128/mSystems.00626-20 (PMC7394361; doi:10.1128/mSystems.00626-20)
Supplement: TABLE S3 [file mSystems.00626-20-st003.docx]

| **Species** | **Group** | **Df** | ***F*** | ***R*^2^** | ***P* value** |
| --- | --- | --- | --- | --- | --- |
| Bg | Skin | 1 | 3.8386 | 0.29899 | 0.008 |
|  | Stomach | 1 | 1.4466 | 0.08292 | 0.045 |
|  | Gut | 1 | 2.0685 | 0.11448 | 0.005 |
| Fl | Skin | 1 | 4.5567 | 0.12815 | 0.001 |
|  | Stomach | 1 | 2.2207 | 0.16797 | 0.001 |
|  | Gut | 1 | 3.2505 | 0.09221 | 0.001 |
| Mf | Skin | 1 | 4.0291 | 0.17496 | 0.001 |
|  | Stomach | 1 | 2.5819 | 0.13185 | 0.001 |
|  | Gut | 1 | 1.4298 | 0.07359 | 0.036 |
| Pn | Skin | 1 | 2.3142 | 0.15111 | 0.004 |
|  | Stomach | 1 | 2.6614 | 0.16993 | 0.002 |
|  | Gut | 1 | 1.4132 | 0.09169 | 0.091 |
| Soil | Soil | 1 | 2.3859 | 0.06058 | 0.003 |
| Water | Water | 1 | 1.8649 | 0.09388 | 0.008 |
