# Supplementary material for: The Behavior of Amphibians Shapes Their Symbiotic Microbiomes
Source: mSystems. 2020 Jul 28;5(4):e00626-20. doi: 10.1128/mSystems.00626-20 (PMC7394361; doi:10.1128/mSystems.00626-20)
Supplement: TABLE S4 [file mSystems.00626-20-st004.docx]

| **Type** | **Df** | **F** | **R^2^** | **P value** |
| --- | --- | --- | --- | --- |
| Skin | 3 | 2.1343 | 0.0777 | 0.001 |
| Stomach | 3 | 2.532 | 0.11074 | 0.001 |
| Gut | 3 | 5.7383 | 0.17008 | 0.001 |
